# Nicolau Syndrome due to Penicillin Injection: A Report of 3 Cases without Long-Term Complication

**DOI:** 10.1155/2016/9082158

**Published:** 2016-11-01

**Authors:** Sara Memarian, Behdad Gharib, Mohammd Gharagozlou, Hosein Alimadadi, Zahra Ahmadinejad, Vahid Ziaee

**Affiliations:** ^1^Growth and Development Research Center, Tehran University of Medical Sciences, Tehran, Iran; ^2^Children's Medical Center, Pediatric Center of Excellence, Tehran, Iran; ^3^Department of Pediatrics, Tehran University of Medical Sciences, Tehran, Iran; ^4^Department of Infectious Diseases, Imam Khomeini Hospital, Tehran University of Medical Sciences, Tehran, Iran; ^5^Pediatric Rheumatology Research Group, Rheumatology Research Center, Tehran University of Medical Sciences, Tehran, Iran

## Abstract

Nicolau syndrome (NS) or livedo-like dermatitis is a rare complication of injection of various medications such as penicillin. The pathophysiology of this events is not clear, but some hypotheses are suggested, such as sympathetic nerve stimulation, embolic occlusion, inflammation, or mechanical injury. In this paper we report 3 cases of NS following benzathine penicillin. Clinical symptoms improved in 2 cases during 2-month follow-up, but one of them had a residual necrosis in the distal phalanges of the toes.

## 1. Introduction

Nicolau syndrome (NS) is a rare condition caused by intramuscular or intra-articular injection of various medications. Several drugs such as penicillin, nonsteroidal anti-inflammatory drugs, corticosteroids, and local anesthetics have been reported to be the cause of NS [1, 2]. The pathogenesis of NS is unknown, but sympathetic nerve stimulation, prostaglandin synthesis block, embolic occlusion, inflammation, and physical obstruction of the blood vessels have been suggested [1]. The disease may have an alarming presenting and makes parents very anxious. Because of its nonspecific signs and symptoms, it could be misdiagnosed as other illnesses such as vasculitis or infectious problems. It may also lead to gangrene of extremities, renal failure, and even death of the patient. Here we present two female children (9 and 2 years old) and one 2-year-old boy, with NS caused by intramuscular injection of benzathine penicillin.

## 2. Case Presentation

### 2.1. Case  1

**Figure 1:**
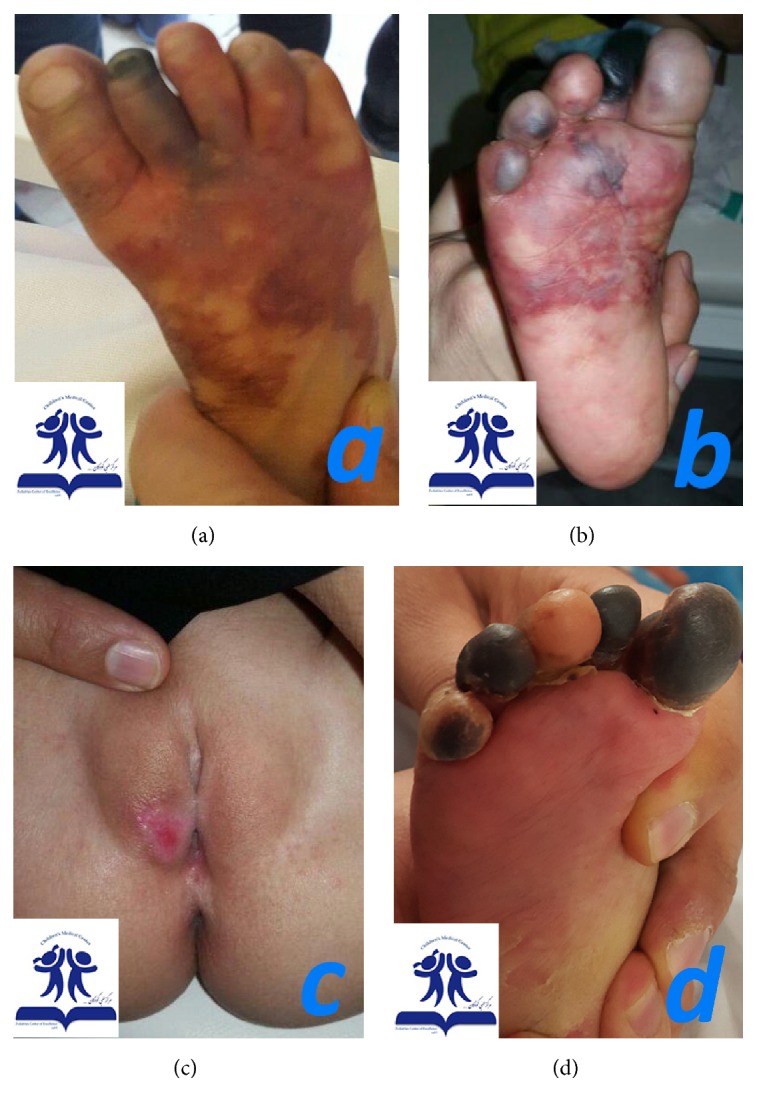
A 2-year-old girl with Nicolau syndrome after injection of benzathine penicillin. (a) The right leg and foot are pale and mottled with ecchymosis and the necrosis of the second toe. (b) Mottling and ecchymosis of the plantar surface and toes of the right foot and the necrotic toes. (c) A round ulcer with about 2-centimeter diameter, on the right labia major. (d) At 2 months' follow-up improvement of the skin ecchymosis was noticed, but a persistent necrosis lesion in the distal phalanges of some of the toes appeared.

A 2-year-old girl referred to Children's Medical Center, Pediatrics Center of Excellence as tertiary referral center in Tehran, Iran, in July 2015 with the complaint of coldness of the right lower limb and mottling and cyanotic patches on lower right thigh, leg, and foot. The problem had started since 3 days ago after she received an injection of benzathine penicillin (600,000 units) to the right buttock, for fever and signs and symptoms of coryza. About half an hour after the injection, the right leg and foot became pale and mottled, and edema and coldness developed few minutes later (Figure 1(a)). The second toe became necrotic (Figure 1(b)). A round ulcer with about 2-centimeter diameter was apparent on the right labia major (Figure 1(c)). There was not any lesion on the site of injection.

She was irritable. The vital signs were stable and she did not have fever. The right leg and foot were tender to touch and she did not allow touching the lower limb and could not bear weight on the right leg. The examinations of the abdomen, heart, respiratory system, and head and neck and other extremities (except the affected limb) were normal. The pulses of the right leg (dorsal pedis, popliteal, and femoral) were examined when she was asleep and were normal and the pulses of both lower limbs were symmetrical. The pulse oximetry (SatpO2) of the right lower limb was 74% and the left was 95%. The right leg and foot were colder; however the pulses of the both lower limbs were symmetrical and normal on palpation. Complete blood cells (CBC), erythrocyte sediment rate, and C-reactive protein levels were within normal ranges. Aspartate aminotransferase, alanine aminotransferase (ALT), creatinine phosphokinase kinase (CPK), and lactate dehydrogenase had been increased about three times of normal but dropped to normal ranges in few days. Coagulation profile, rheumatologic tests, and color Doppler ultrasonography of lower limb arteries and veins were normal. The patient was prescribed nifedipine tablet 5 milligrams daily and Ibuprofen syrup every 8 hours. The genital ulcer was treated with daily dressing. During the admission, the patient's irritability improved and the mottled purple patches faded. The patient was discharged with the prescription of nifedipine tablet 5 milligrams daily, prednisolone tablet 2.5 milligrams three times a day, and Ibuprofen syrup 3.5 milliliters in case of pain. At 2 months' follow-up lesions were significantly improved, with a similar color and temperature in the whole foot except for a persistent necrosis in the distal phalanges of some of the toes (Figure 1(d)). Vulvar lesion improved completely without any scar lesion.

### 2.2. Case  2

**Figure 2:**
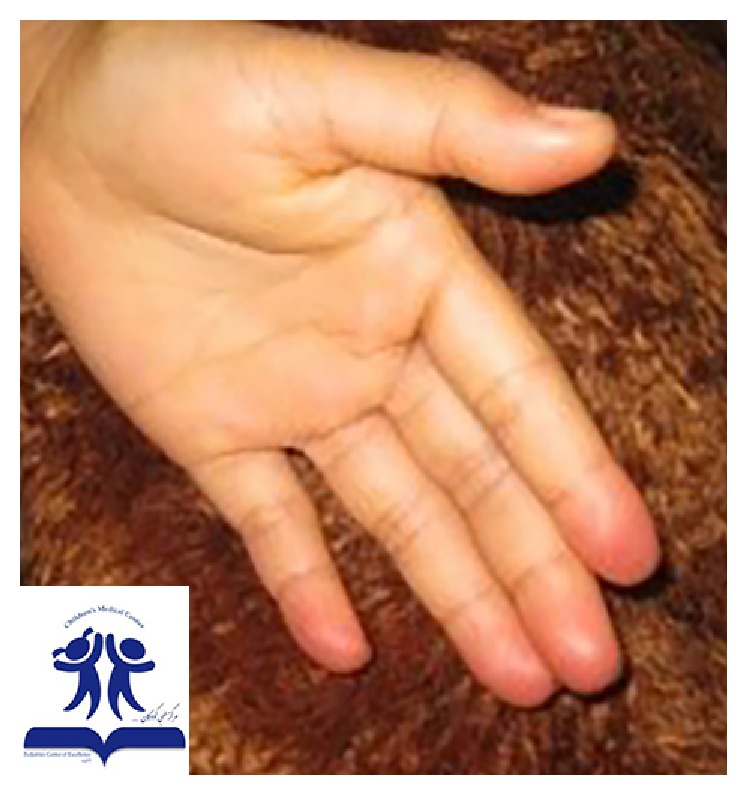
A 9-year-old girl with Nicolau syndrome after injection of benzathine penicillin in the deltoid muscle. Hyperemia and redness of the tips of the fingers of the left hand.

A nine-year-old girl presented to our referral rheumatologic clinic with the presentation of mottled patch on the lateral side of the left arm. She had pain, edema, and tenderness on the left arm, forearm, and hand. The distal phalanxes of all left fingers were cold and cyanotic. The problem had started after she received an injection of benzathine penicillin 600,000 units to the left deltoid muscle. About 3 hours after the injection, she had 3 vomiting episodes and edema and felt lethargic; paleness and mottling of the left upper limb developed gradually. The tips of the fingers of the left hand became hyperemic and red (Figure 2).

The pulses of the limb were normal and symmetrical to the right upper limb. The motor exam was normal, but there was some mild paresthesia in the route of the ulnar nerve of the affected limb. There was not any sign of compartment syndrome in the affected limb and a color Doppler ultrasonography revealed normal blood flow in the veins and arteries of the left upper limb. The examinations of the other systems were normal and the vital signs were stable. Complete blood cells (CBC), erythrocyte sediment rate, C-reactive protein levels, and coagulation profile tests were within normal ranges.

She was administered an intravenous injection of Hydrocortisone 100 mg stat, enoxaparin 20 units subcutaneously every 12 hours, ointment of trinitroglycerin on the affected fingers, and analgesic. During admission, the signs and symptoms improved gradually. She was discharged in few days. On the follow-up visits after 1 month there were no residual clinical findings.

### 2.3. Case  3

**Figure 3:**
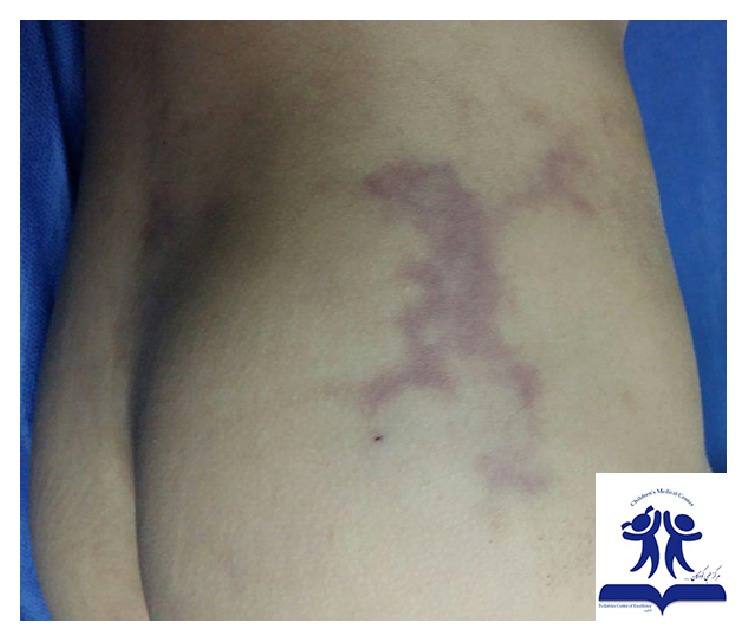
A 2.5-year-old boy with Nicolau syndrome after injection of benzathine penicillin. Ecchymosis in the site of injection with pain and coldness in the distal of injection.

A 2.5-year-old boy (who had been under observation for PFAPA (periodic fever, adenitis, pharyngitis, aphthous ulcer)) was brought to our clinic with pain and coldness of his right lower limb and inability of weight bearing on the affected leg, soon after injection of 600,000 units of intramuscular benzathine penicillin. Penicillin was prescribed for patient by a general practitioner during an episode of fever with diagnosis of streptococcal pharyngitis. There is ecchymosis in the site of injection (Figure 3). The blood pressure and pulses of the affected limb were normal. The blood and urine lab workups were within normal except an erythrocyte sediment rate (ESR) of 48 mm/h which was measured before the injection. A Doppler ultrasonography of the arteries and veins and the ultrasonography of the hip were also reported normal. By applying conservative measures and warm compression and massage therapy, after 72 hours of the admission, his condition started to improve and the skin's color and temperature changed to normal gradually without prescribing any medication. He achieved the ability of bearing weight on the affected limb and walking again. On the follow-up visit after 1 month, he did not seem to have any new clinical finding.

## 3. Discussion

Nicolau syndrome (NS) which has also been mentioned as “livedo-like dermatitis” and “embolia cutis medicamentosa” is a rare condition caused by intramuscular or intra-articular injection of various medications and it was described for the first time in 1924 and 1925 by Freudenthal and Nicolau after an intramuscular (gluteal) injection of bismuth for the treatment of syphilis [7, 12]. Several drugs such as penicillin, nonsteroidal anti-inflammatory drugs, corticosteroids, and local anesthetics have been reported to be the cause of NS [1, 4, 13–15]. Intramuscular injection of Phenobarbital, Chlorpromazine, Gentamicin, Dexamethasone, DPT vaccine, Diphenhydramine, and lidocaine has also led to NS. In severe cases, NS may lead to death [2]. NS is more prevalent in children especially in those under 3 years old. The event of artery embolism could be more likely in younger children because of the smaller vascular size [1]. Saputo and Bruni reviewed 102 cases of NS and have shown that 78.43% of cases occurred under 12 years old [8]. One theory explains that NS happens when an intramuscular drug is injected into an artery and causes thrombosis and muscle and subcutaneous necrosis. However the pathogenesis of NS is unknown, but the mechanisms have been suggested in Table 1.

One important clinical element is the sudden onset with relation to the injection, often with no lesion at the injection site. The signs of NS are skin discoloration, intense pain, and inflammation. Necrosis usually comes after hyperemia, discoloration of skin, formation of hemorrhagic patch at the site of injection, and livedoid dermatitis. Local vasospasm causes pallor [1]. One-third of the patients may experience neurologic complications (usually transitional) which are most frequently hypoesthesia and paraplegia [16]. NS has also been reported to cause compartment syndrome of the limb, hyperkalemia, renal failure, and death [2]. Paralysis of the lower limb can happen and can be explained by medication embolism. Embolus in the vessels of gluteal muscle can reach the internal iliac artery and then vertebral canal by retrograde flow. This arterial stenosis can result in peripheral neve disturbance and lower limb paralysis [17].

There are no definite criteria for diagnosis of this syndrome and diagnosis was made according to clinical symptoms in a patient with history of recent injection and after exclusion of other similar disorders. The main differential diagnosis of NS is necrotizing fasciitis. History of recent trauma and injury, illness, local tenderness, and air trapping in the involved tissue are expected in the necrotizing fasciitis [18]. Vasculitis and cutaneous embolization of cardiac myxoma are other differential diagnoses of NS [19]. A recent history of injection in the proximal of the involved limb is a good diagnostic clue in NS.

Administration of anticoagulants, intravenous corticosteroids and vasoactive therapy (like pentoxifylline) is reported to support the improvement of the patients [15, 20]. In some cases, fasciotomy was performed for compartment syndrome [4, 14]. Inhibition of phosphodiesterase by pentoxifylline may relieve vasospasm. Topical corticosteroids may help in the improvement of tissue inflammation [2]. The wounds may be treated by antibiotics, surgical debridement, dressing, and skin graft [1]. The correct method of intramuscular injection (injection in the supralateral part of the gluteal muscle, long enough needle to reach the muscle, Z-track injection) can minimize the risk of NS [21]. Aspirating the syringe prior to the injection and never injecting more than 5 milliliters of drug at one time and one site while using the Z-track method are other precautions that may prevent the risk of NS [6, 21]. The benzathine benzylpenicillin injection is a condensed suspension and it may hinder the blood on aspirating into the syringe [4, 13–15]. Pain at the injection site, skin discoloration, and temperature changes had been the most obvious and common symptoms in our patients. We did not see catastrophic complications such as acute renal failure, hyperkalemia, limb amputation, or disfiguring features in our patients and all of them recovered by simple treatment measures in quite short period of time. One of them (the 9-year-old girl) had received the injection in the inappropriate site (penicillin in the deltoid muscle). This issue reminds all of the physicians and medical staff of the importance of the correct injection method and the right prescription of antibiotics and medications.

## Figures and Tables

**Table 1:** Different theories for pathogenesis of the Nicolau syndrome.

Sympathetic nerve stimulation [1, 3]	The sympathetic nerve stimulation by pain (from the periarterial or intra-arterial injection) causes vasospasm and ischemia
Prostaglandin synthesis block [2, 4–6]	Pharmacologic characteristics of NSAIDs which block prostaglandin synthesis cause vasospasm and ischemia
Embolic occlusion [7–9]	Accidental intra-artery injection causes embolic occlusion of the arteries
Inflammation [5, 10]	Perivascular inflammation caused by cytotoxic reaction to the injected medication
Mechanical injury [2, 5, 11]	Physical obstruction of the blood vessels caused by the lipophilic drugs which penetrated the vessels
